# The influence of selected factors on leaching of metals from sewage sludge

**DOI:** 10.1007/s11356-018-3094-8

**Published:** 2018-09-25

**Authors:** Monika Janas, Alicja Zawadzka, Robert Cichowicz

**Affiliations:** 1 Faculty of Process and Environmental Engineering, Lodz University of Technology, Wolczanska 213, 90-924 Lodz, Poland; 2 Faculty of Architecture, Civil and Environmental Engineering, Lodz University of Technology, Politechniki 6, 90-924 Lodz, Poland

**Keywords:** Sewage sludge, Metals, Tessier procedure, Sequential extraction, Composting, Calcium oxide

## Abstract

In Poland, the amount of municipal sewage sludge that contains both organic and inorganic pollutants increases steadily. As a result of penetration of atmospheric precipitations through sludge layers, products of biochemical decomposition of organic matter and soluble mineral compounds are washed away and form contaminated leachates (Arain, J. Hazard. Mater. 154:998–1006, 2008; Fang, J. Hazard. Mater. 310:1–10, 2016; Ignatowicz, Environ. Res. 156:19–22, 2017). Metals contained in these leachates may be particularly burdensome and dangerous, which is due to the toxic nature that disturbs the natural biological balance (Fytili and Zabaniotou, Renew. Sust. Energy Rev. 12 (1): 116–140, 2008). In order to check bio-availability of metals in sewage sludge and find out resulting risks to the environment and human health, apart from the determination of total metal content, speciation analysis is often used. It makes possible a quantitative determination of various chemical forms of metals which are bound in the sewage sludge and finding which of them poses the greatest threat to the environment (Amir, Chemosphere 59:801–810, 2005; Ciba, Waste Manage. 23:897–905, 2003; Hei, Procedia Environ Sci 31:232–240, 2016; Liu, Chemosphere 67(5):1025–1032, 2007).

The degree of immobilization of selected metals in sewage sludge has been determined using one of the sequential extraction methods designed to identify groups of compounds with which the metal is bound. Such a method is the Tessier procedure (Janas, Pol J Environ Stud 26(5A):37–41, 2017). Results of this research were used to assess the threat resulting from the increase in the amount of sewage sludge; the management of which is subject to constant restrictions (storage of sewage sludge has been prohibited since January 1, 2016).

As a result of the conducted research, it was found that metals in sewage sludge, which undergo various transformations, are very difficult to immobilize. The addition of calcium oxide and an agent supporting the composting process to the sludge does not affect radically the increase of leaching of the analyzed elements from the sludge.

**Graphical abstract:**
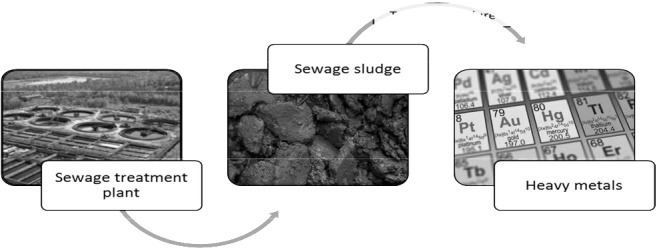
The schema of proceedings in leaching of heavy metals from sewage sludge after various modifications.

The schema of proceedings in leaching of heavy metals from sewage sludge after various modifications.

## Introduction

Metals toxicity present in the environment attracts the interest of many researchers. Metals in the environment may be both of natural origin and can result from anthropogenic activity. Sludge generated during wastewater treatment may contain environmentally harmful and toxic compounds. Particularly dangerous are persistent metals. These elements are not degraded and accumulated in the environment (Alvarez et al. 2002; Liu et al. 2007; Tessier et al. 1997; Wang et al. 2005). Metals in trace amounts are necessary for life, but their excess or disturbed proportions have negative influence on plants, animals, and human health. Plants are the main recipients of minerals from the soil and at the same time the basic source of nutrition for animals and people. Metals in trace amounts are strongly sorbed by components of solid soil phase. They are poorly leached and do not move easily in the soil profile. After exceeding the permissible content in the ground, metals reduce soil fertility and inhibit the enzymatic activity of plants growing on it (Arain et al. 2008; Ignatowicz 2017; Lasheen and Ammar 2009). In this respect, the most important are zinc, copper, cadmium, and lead, due to their phytotoxicity, and nickel which is accumulated mainly in the kidneys causing allergic reactions and diseases of kidneys, lungs, and liver (Arain et al. 2008).

Source of metals in sewage sludge is industrial wastewater and surface runoff. Approximately, 80 to 90% of metals contained in wastewater are accumulated in sewage sludge. The content of individual elements may vary depending on the wastewater origin. Industrial sectors that can significantly increase the content of metals in sewage sludge include metallurgy and metalworking industry, machine industry, leather tanning, chemical, as well as pulp and paper industries (Amir et al. 2005; Ciba et al. 2003; Liu et al. 2007). The main industrial branches involved in direct and indirect contamination with metals are listed in Table 1 (Liu et al. 2007).

**Table 1:** Selected metals and industrial sources of their emission to the environment

Metals	Industrial branches
Cadmium (Cd)	Electroplating, production of dyes, batteries, accumulators, paints and plastics, polymer stabilizers, chemical industry, graphic and printing plants
Lead (Pb)	Production of dyes, accumulators, batteries, fertilizers, energy and electrochemical industry
Chromium (Cr)	Galvanizing, tanning and textile industry, dyes and plastics production, wood impregnation, printing and graphic plants
Copper (Cu)	Metallurgy, dyes and textile industries, production of plant protection products and fertilizers
Mercury (Hg)	Production of batteries, phosphoric acid, caustic soda, pulp mills, production of plant protection products
Nickel (Ni)	Galvanizing industry, paper industry, fertilizer factories
Zinc (Zn)	Production of batteries, paints, plastics, polymer stabilizers, textile industry, printing and graphic plants

In sewage sludge, metals may be in a dissolved or precipitated form and also in the co-precipitated form with metal oxides. They may occur as oxides, hydroxides, phosphates, sulfides, sulfates, silicates, and organic combinations in the form of complexes. Chemical forms of metals accumulated in sewage sludge are characterized by different mobility (Carlson and Morrison 1992; Fuentes et al. 2008). In order to determine the behavior of metals under environmental conditions and their impact on organisms living in a given ecosystem, in addition to determining their total content, their leaching rate should be assessed. Such an assessment can be made by determining the amount of metals bound by individual components (fractions) of sewage sludge. A method commonly used for this purpose is the sequential analysis (Carlson and Morrison 1992; Fuentes et al. 2008; Lasheen and Ammar 2009; Tessier et al. 1997; Zhu et al. 2011). The literature describes a number of sequential extraction methods and their modifications; however, the Tessier method is the best known. It is used to separate exchangeable, carbonate, reducible, oxidable (bound with organic matter), and residual metal fractions (Fuentes et al. 2008; Janas et al. 2017).

The subject of the research was sewage sludge in which the mobility of metals was determined and the influence of sludge composting with the addition of calcium oxide and a commercial preparation supporting the process on the degree of leaching of metals was investigated.

## Tested material

The experimental material was sewage sludge obtained from the Combined Sewage Treatment Plant in Lodz with a capacity of up to 215.3 thousand m^3^/day. The Combined Sewage Treatment Plant of Lodz agglomeration is a typical biomechanical treatment plant with increased removal of biogenic compounds. In the plant, where over half of all municipal sewage generated in the Lodz Region is treated, a three-phase activated sludge process, a so-called MUCT process, is used. The treatment plant load expressed as an equivalent number of inhabitants is 1,026,260 M.

Two types of sewage sludge were used in the research. The sludge was collected in accordance with PN-EN ISO 5667-13:2011 standard from digesters and after being concentrated on belt thickeners. Sludge samples were collected throughout the week (7 days) to reflect changing weather conditions. A single sludge sample weighed approximately 1.5 kg and was an averaged representative sample. Basic physicochemical properties of the sludge collected are presented in Table 2.

**Table 2:** Basic physicochemical properties of the tested sewage sludge

Parameter tested	Fermented sludge	Dewatered sludge
Odor	Earthy	Earthy
Form	Clammy	Lumpy
Color	Black gray	Brownish
pH	8.7 ± 0.1	9.1 ± 0.1
Total nitrogen % dm	6.6 ± 0.9	6.7 ± 0.9
Total phosphorus % dm	1.6 ± 0.2	1.8 ± 0.2
Organic substances % dm	66.6 ± 9.5	70.7 ± 10.5
Dry matter of sludge %	5.1 ± 0.7	20.4 ± 3.5
Zinc mg/kg dm	721 ± 108	865.2 ± 130
Lead mg/kg dm	13.4 ± 2.1	16.6 ± 2.5
Cadmium mg/kg dm	1.12 ± 0.2	1.35 ± 0.2
Chromium mg/kg dm	9.4 ± 1.2	11.3 ± 1.7
Copper mg/kg dm	44.8 ± 6.0	53 ± 7.4
Nickel mg/kg dm	7.3 ± 1.0	8.8 ± 1.3
Arsenic mg/kg dm	4.18 ± 0.6	5.2 ± 0.8
Mercury mg/kg dm	0.95 ± 0.1	1.14 ± 0.1

Sludge samples were mixed separately in different proportions with calcium oxide and a substance supporting the process of composting. The resulting mixtures were conditioned for 7 days, and next some samples were subjected to additional composting in 100-dm^3^ plastic containers for 3 months at a temperature of about 20 °C. During the composting, the sludge was stirred every 30 days. The study covered 15 experiments in three replications:

1. Dewatered sewage sludge,
2. Dewatered sludge with the addition of 1.5-g CaO,
3. Dewatered sludge with the addition of 15-g CaO,
4. Dewatered sludge with the addition of 30-g CaO,
5. Dewatered sludge with the addition of 75-g CaO,
6. Digested sludge,
7. Digested sludge with the addition of 1.5-g CaO,
8. Digested sludge with the addition of 7.5-g CaO,
9. Digested sludge with the addition of 15-g CaO,
10. Digested sludge with the addition of 30-g CaO,
11. Dewatered sludge after composting,
12. Dewatered sludge after composting with the addition of 3-g agent supporting the process,
13. Dewatered sludge after composting with the addition of 30-g agent supporting the process,
14. Dewatered sludge after composting with the addition of 15-g CaO and 3-g agent supporting the process,
15. Dewatered sludge after composting with the addition of 30-g CaO and 3-g agent supporting the process.

## Research methodology

In the analyzed sewage sludge, the total content of selected metals was determined, and metal fractions were specified according to the method proposed by the Tessier. Before the proper sequential analysis, the experimental material was pretreated. The sewage sludge was dried in a laboratory dryer at a temperature of 105 °C until obtaining a constant mass. In order to get reliable samples, the dried sewage sludge was ground in a mortar, and for each experiment, three samples 20 g each were weighed.

The so prepared sewage samples were subjected to the extraction analysis according to the Tessier procedure, which enabled a stepwise separation of FIDE metal fractions released in different conditions due to leaching with solutions of growing leaching power. As a result, the following fractions were determined:

- F – I Ion-exchange fraction – the material samples were leached at room temperature using 40-ml solution of magnesium chloride (MgCl_2_) at a concentration of 1 mol/dm^3^ and pH = 7. The samples were stirred for 1 h using a magnetic stirrer.
- F – II Carbonate fraction – the residue of fraction I was leached at room temperature with 40-ml solution of sodium acetate (CH_3_COONa) at a concentration of 1 mol/dm^3^, while maintaining continuous stirring with a magnetic stirrer for 5 h.
- F – III Hydroxyl fraction – the residue of fraction II was extracted with 100-ml solution of 0.04-mol/dm^3^ hydroxylamine hydrochloride (NH_2_OH∙HCl) in 25% acetic acid (CH_3_COOH). To ensure a proper process temperature (96 ± 3 °C), the flask with the sample was placed in a water bath for 6 h and stirred occasionally.
- F – IV Organic fraction – to the residue of fraction III placed in a round-bottom flask, a system of coolers was connected, 15-ml solution of nitric acid (HNO_3_) at a concentration of 0.02 mol/dm^3^ and 25-ml 30% hydrogen peroxide (H_2_O_2_) was added successively. The mixture was heated at a temperature of 85 ± 2 °C for 2 h, stirring occasionally.
- F – V Residual fraction – the residue of fraction IV was taken up in a mixture of concentrated hydrochloric and nitric acids (V) (the “Aqua regia”) in a volume ratio of 3:1 in an amount of 25 ml. The process of thermal mineralization was carried out in a water bath using a system of back-pressure coolers for 2 h, while ensuring the process temperature of 85 °C (Lasheen and Ammar 2009; Tessier et al. 1997).

After each extraction step, the solid phase was separated from liquid using a filtration kit. The resulting extract was quantitatively transferred to plastic containers that have been properly marked. Additionally, the residue after extraction was washed every time with 40 ml of distilled water, and the resulting filtrate was discharged. Between particular leaching series, the filters with sludge were dried in a laboratory dryer for about a day at a temperature of 105 °C.

The content of metals in the extracts was determined according to ISO 9001:2000 using a Perkin-Elmer atomic absorption spectrophotometer 3100 AAS-BG in three separate sewage sludge samples.

## The results and discussion

Table 3 presents content of individual metal forms in sewage sludge in relation to the total content in accordance with the Tessier procedure.

**Table 3:** Speciation of metal forms in sewage sludge according to the Tessier procedure

Experiment no.	Fraction	Metal							
		Lead	Chromium	Cadmium	Copper	Nickel	Mercury	Zinc	Arsenic
		mg/kg dm	mg/kg dm	mg/kg dm	mg/kg dm	mg/kg dm	mg/kg dm	mg/kg dm	mg/kg dm
1	Ion-exchange	3.3	1.3	0.2	16.4	2.5	0.3	156.5	1.2
	Carbonate	5.0	2.2	0.4	3.3	0.9	0.2	238.8	1.4
	Hydroxyl	2.5	2.1	0.2	5.2	1.3	0.3	158.3	1.1
	Organic	4.9	3.1	0.3	14.1	2.3	0.1	112.9	0.7
	Residual	0.5	2.6	0.1	13.5	1.7	0.1	192.7	0.6
2	Ion-exchange	3.6	1.3	0.3	14.9	2.5	0.3	142.2	1.2
	Carbonate	4.6	2.1	0.1	9.3	0.7	0.2	181.7	1.2
	Hydroxyl	2.1	1.9	0.2	3.7	1.2	0.3	182.3	0.9
	Organic	4.2	2.9	0.2	12.7	3.0	0.1	130.0	0.6
	Residual	1.7	2.8	0.4	12.0	1.4	0.1	225.0	1.2
3	Ion-exchange	1.3	1.2	0.2	12.1	0.6	0.1	193.3	0.8
	Carbonate	5.8	2.0	0.2	4.8	2.3	0.2	171.9	1.5
	Hydroxyl	2.2	1.9	0.3	9.0	0.6	0.3	187.9	1.1
	Organic	5.4	3.0	0.2	11.9	1.8	0.2	176.5	0.8
	Residual	1.5	3.2	0.3	15.0	3.3	0.1	131.6	0.8
4	Ion-exchange	1.6	1.1	0.1	13.1	1.6	0.3	218.6	1.0
	Carbonate	2.6	1.4	0.2	6.4	1.8	0.3	112.7	1.1
	Hydroxyl	4.7	2.0	0.3	7.0	1.5	0.2	200.0	1.1
	Organic	2.6	2.6	0.2	14.0	0.5	0.2	190.7	1.0
	Residual	4.8	4.0	0.4	12.0	3.4	0.2	132.1	1.0
5	Ion-exchange	1.4	1.7	0.3	7.5	2.4	0.2	250.0	1.3
	Carbonate	1.2	1.5	0.3	6.4	1.6	0.3	164.2	1.0
	Hydroxyl	1.7	3.1	0.1	7.3	1.7	0.2	161.5	0.7
	Organic	1.3	1.4	0.1	15.6	1.7	0.2	143.7	0.7
	residual	10.6	3.5	0.5	16.1	1.3	0.1	141.7	1.3
6	Ion-exchange	3.1	1.0	0.3	19.5	1.6	0.3	187.4	1.1
	Carbonate	4.7	1.2	0.2	8.2	1.1	0.2	148.1	1.0
	Hydroxyl	1.3	2.1	0.1	6.2	0.8	0.1	142.1	0.6
	Organic	2.8	3.3	0.1	4.0	1.1	0.1	88.6	0.4
	Residual	1.3	1.6	0.3	6.7	2.7	02	149.7	0.8
7	Ion-exchange	3.1	1.0	0.3	22.5	1.6	0.3	177.7	1.0
	Carbonate	4.2	1.3	0.3	4.3	1.2	0.2	150.6	0.9
	Hydroxyl	1.2	2.1	0.1	7.1	1.0	0.2	141.5	0.8
	Organic	2.5	3.3	0.1	4.2	1.1	0.1	88.4	0.4
	Residual	2.1	1.6	0.2	6.5	2.3	0.2	157.8	1.0
8	Ion-exchange	2.3	0.7	0.2	12.5	1.4	0.3	213.9	1.1
	Carbonate	4.3	0.9	0.1	10.6	1.1	0.2	193.4	0.8
	Hydroxyl	1.0	2.0	0.2	9.4	0.5	0.1	122.7	0.8
	Organic	2.5	2.8	0.2	4.3	1.0	0.1	115.8	0.6
	Residual	2.0	2.9	0.4	7.7	3.4	0.2	71.1	0.7
9	Ion-exchange	1.3	1.3	0.2	6.1	1.2	0.3	161.4	1.2
	Carbonate	4.9	2.0	0.2	12.3	1.0	0.2	177.4	0.9
	Hydroxyl	1.1	0.9	0.2	10.0	1.1	0.2	162.7	0.7
	Organic	3.1	2.2	0.1	6.1	0.7	0.1	117.6	0.6
	Residual	2.8	2.9	0.3	10.2	3.3	0.1	95.9	0.6
10	Ion-exchange	1.7	1.7	0.2	12.0	1.0	0.3	181.9	1.1
	Carbonate	3.8	2.6	0.2	7.6	0.9	0.2	164.8	0.9
	Hydroxyl	1.4	1.4	0.1	6.8	0.9	0.1	153.9	0.7
	Organic	1.2	1.3	0.1	3.7	0.8	0.1	97.0	0.5
	Residual	5.1	2.2	0.4	14.5	3.6	0.1	117.5	0.7
11	Ion-exchange	3.6	2.0	0.3	16.7	1.1	0.2	220.3	1.3
	Carbonate	3.2	2.1	0.2	8.4	1.7	0.2	137.6	1.0
	Hydroxyl	0.7	1.8	0.1	8.3	1.4	0.2	140.9	0.7
	Organic	3.8	1.2	0.2	8.9	1.6	0.1	173.1	0.6
	Residual	5.1	4.1	0.5	10.6	2.7	0.3	191.3	1.5
12	Ion-exchange	4.8	1.1	0.2	9.5	2.3	0.3	135.7	1.2
	Carbonate	4.3	2.6	0.2	8.7	1.0	0.3	137.9	1.2
	Hydroxyl	0.7	1.7	0.1	9.1	1.1	0.1	140.0	0.7
	Organic	2.7	1.4	0.1	10.0	2.2	0.1	146.4	0.8
	Residual	3.9	4.4	0.7	15.5	2.2	0.2	300.0	1.2
13	Ion-exchange	4.6	3.4	0.3	10.8	2.2	0.3	182.2	1.3
	Carbonate	6.2	2.3	0.3	12.3	0.9	0.3	150.7	1.4
	Hydroxyl	1.1	1.9	0.2	9.8	1.3	0.1	192.2	0.7
	Organic	0.7	1.3	0.1	8.6	1.4	0.1	96.1	0.7
	Residual	3.9	2.1	0.4	11.3	2.8	0.2	238.0	0.8
14	Ion-exchange	6.4	3.0	0.1	15.3	1.8	0.3	178.0	1.4
	Carbonate	2.5	2.5	0.1	12.7	0.7	0.3	17.6	1.2
	Hydroxyl	2.2	1.8	0.1	11.4	0.6	0.2	158.5	0.8
	Organic	2.0	1.6	0.2	0.2	2.3	0.2	110.3	0.7
	Residual	3.2	2.1	0.7	13.2	3.2	0.1	235.7	1.1
15	Ion-exchange	6.6	3.5	0.1	11.0	1.1	0.3	133.7	1.5
	Carbonate	1.7	2.2	0.2	24.0	1.2	0.2	160.2	1.0
	Hydroxyl	1.6	2.6	0.2	0.3	1.0	0.2	133.0	0.7
	Organic	0.7	1.7	0.3	0.2	0.6	0.2	107.6	0.6
	Residuzal	5.8	1.1	0.5	16.5	4.9	0.2	324.7	1.1

The results of metal fractionation in sewage sludge and compost are shown in Figs. 1, 2, 3, 4, 5, 6, 7, and 8. The number of experiments and marked fractions correspond to the description presented in the experimental section. The sequential analysis showed that sewage sludge contained various forms of metals.

**Fig. 1:**
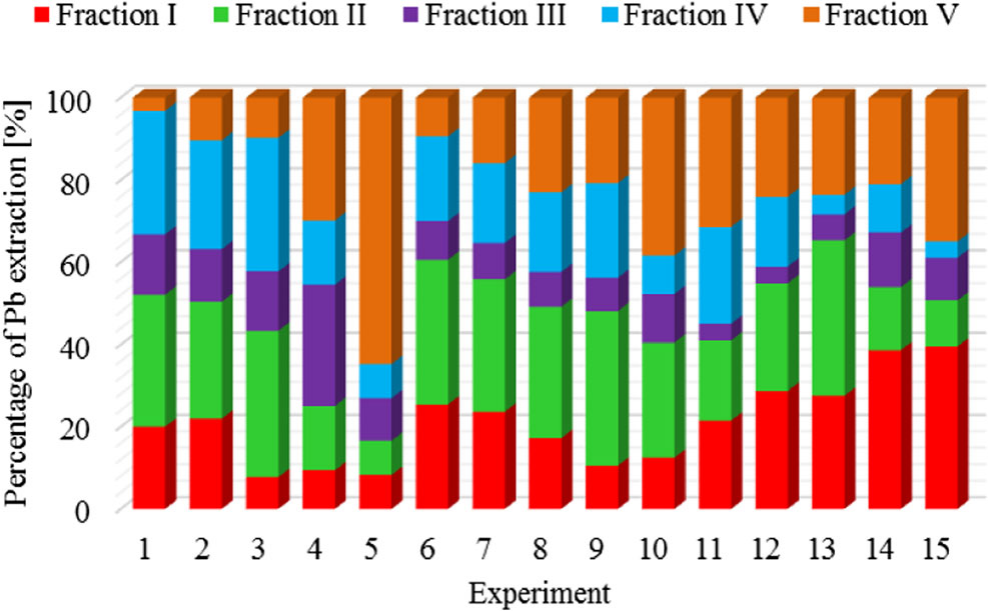
The amount of leached lead (Pb) in all fractions and experiments

**Fig. 2:**
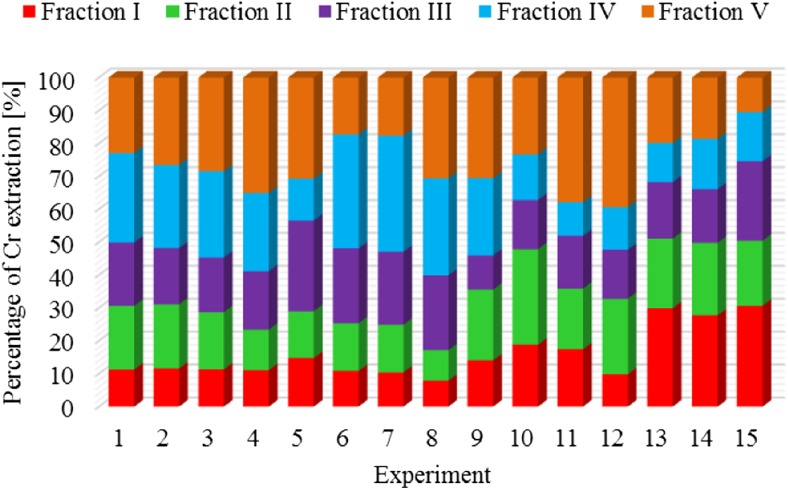
The amount of leached chromium (Cr) in all fractions and experiments

**Fig. 3:**
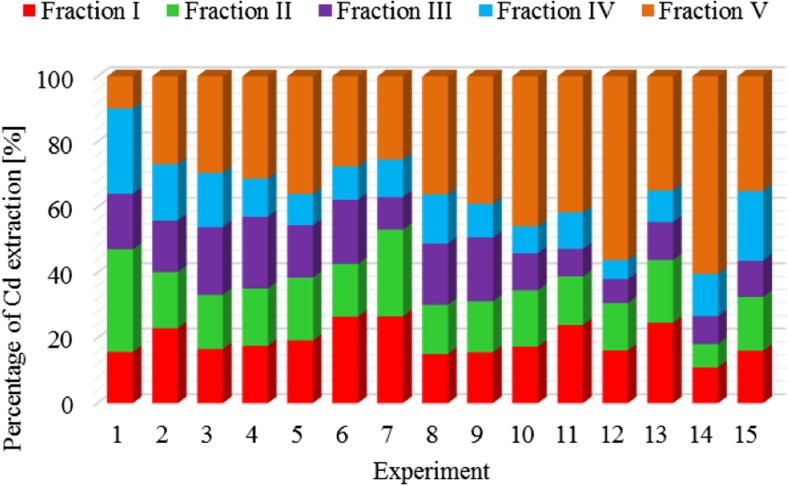
The amount of leached cadmium (Cd) in all fractions and experiments

**Fig. 4:**
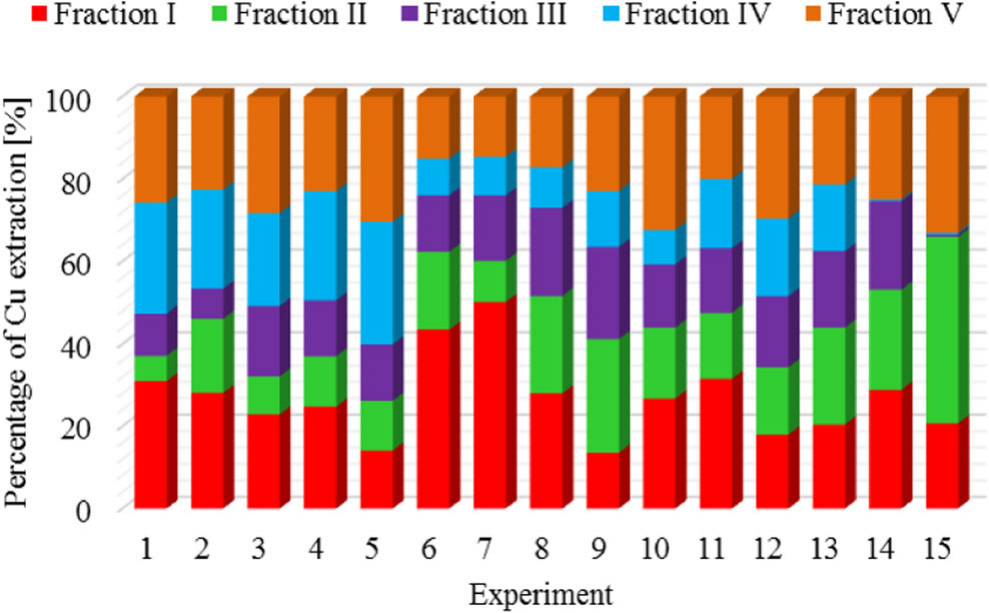
The amount of leached copper (Cu) in all fractions and experiments

**Fig. 5:**
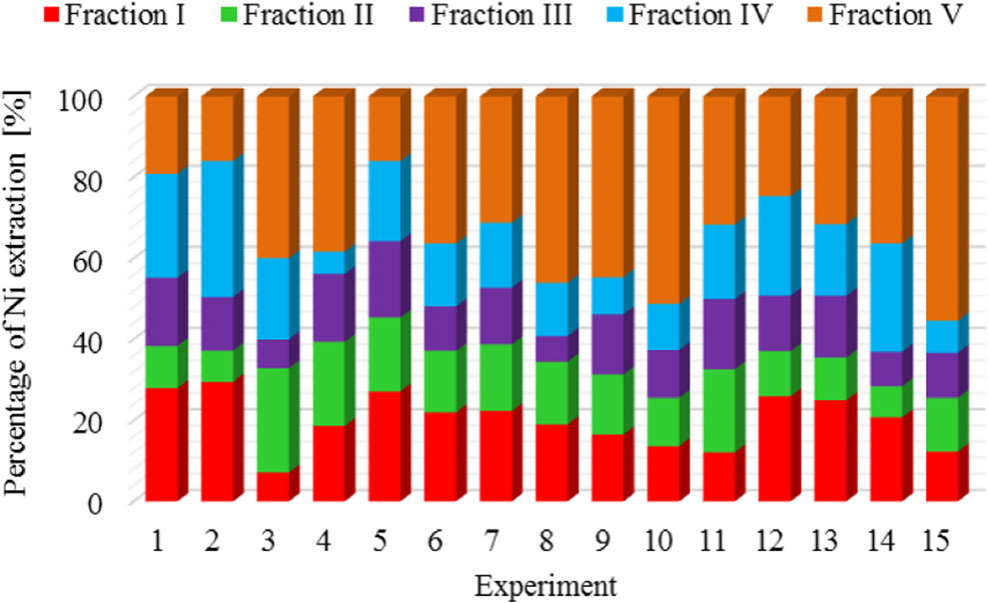
The amount of leached nickel (Ni) in all fractions and experiments

**Fig. 6:**
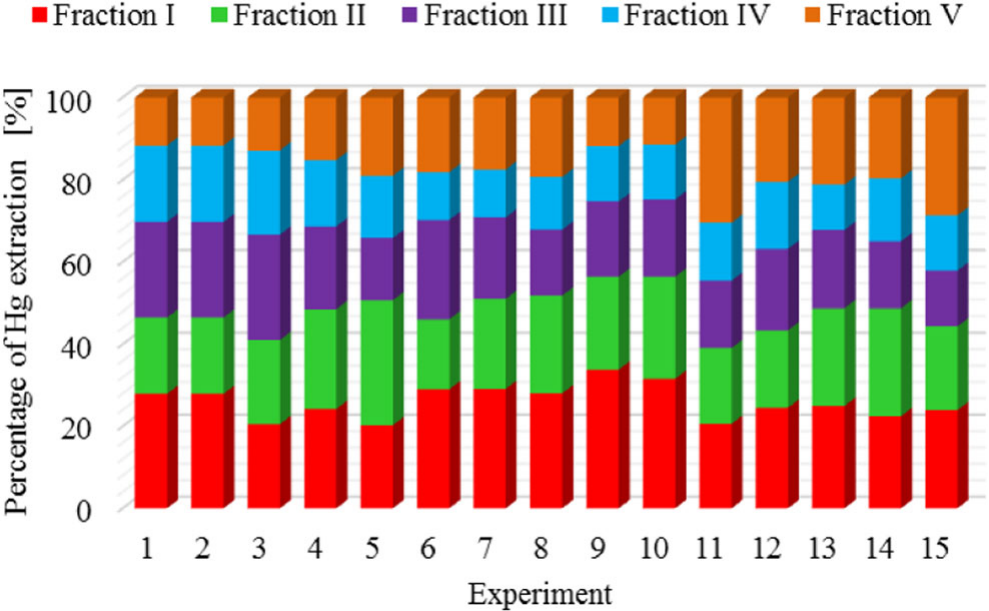
The amount of leached mercury (Hg) in all fractions and experiments

**Fig. 7:**
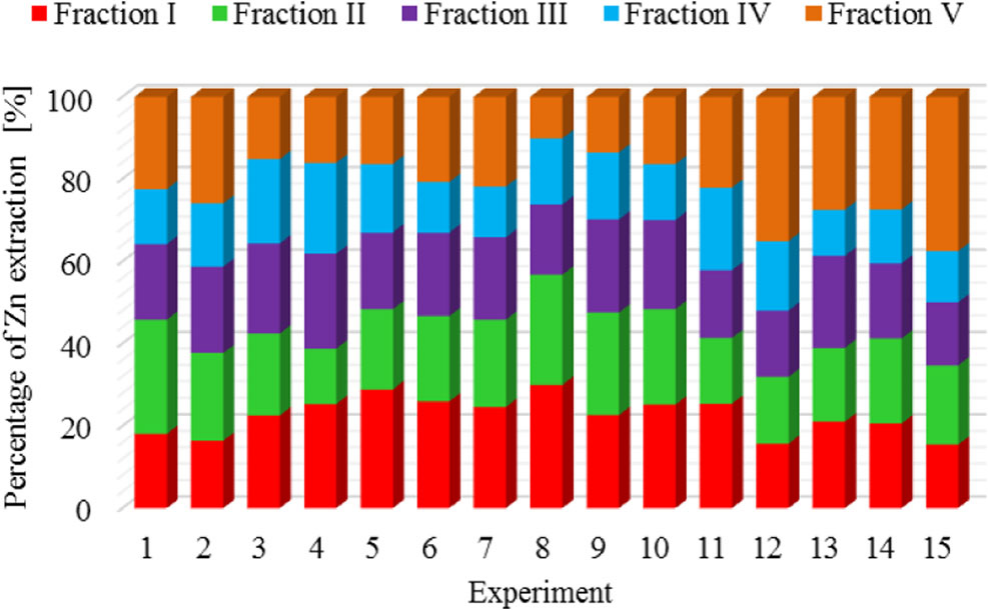
The amount of leached zinc (Zn) in all fractions and experiments

**Fig. 8:**
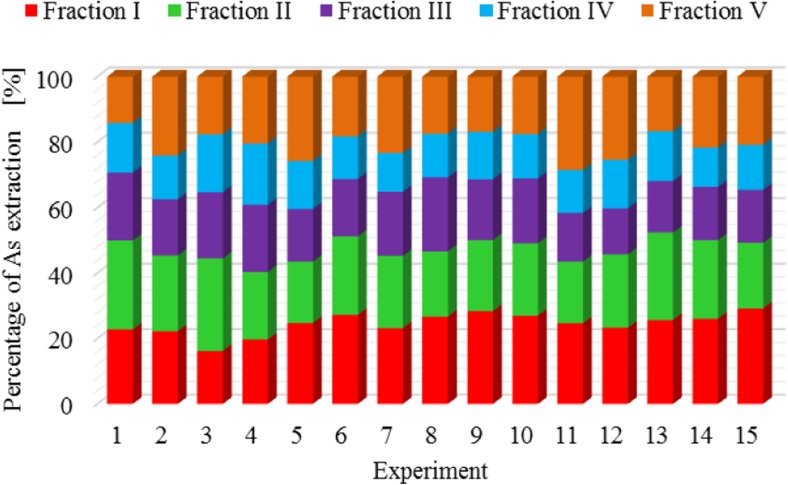
The amount of leached arsenic (As) in all fractions and experiments

In the case of lead, the lowest degree of leaching this metal from the sewage sludge is observed in experiment no. 5, in which for stabilization of the dewatered sludge as much as 75 g of calcium oxide was used. In contrast, this element is most effectively leached from the dewatered sludge not subjected to any treatment.

Another tendency is observed in the case of chromium, as in the dewatered sludge with the addition of 30-g calcium oxide and 3 g of a substance supporting the composting process; the degree of leaching of this element is the highest, while the lowest leaching degree is obtained in experiment no. 12 (the dewatered sludge is composted only with the addition of 3 g of the substance supporting the process).

In the case of cadmium, the highest leaching is observed in experiment 1, i.e., when the analysis concerned the dewatered sludge without any additional treatment. The lowest leaching of this metal in the sludge was obtained in experiment 14, where the agent stabilizing the dewatered sludge subjected to the composting process was 15 g of calcium oxide and 3 g of substance supporting this process.

The results of the analysis of copper leached from sewage sludge and compost showed very interesting circumstances. In experiments 14 and 15, the highest degree of leaching was observed in fractions I–III, while in fraction IV, it was negligible. In both experiments, the experimental material was sludge composted with the addition of 15 g and 30 g of calcium oxide, respectively.

A detailed analysis of the results obtained for nickel shows that this metal is leached most effectively in the sludge which was not subjected to any additional transformations or underwent very slight changes, i.e., experiments 1 and 2. Again, the lowest degree of leaching was obtained in experiment 15, in which the analysis comprised dewatered sludge composted with the addition of 30-g CaO and 3 g of substance supporting the process.

In the case of mercury, it is not possible to specify the experiment in which the highest or lowest leaching of this element was clearly observed. Results of the analysis of mercury content in subsequent fractions are very similar. A similar tendency is observed in the case of zinc and arsenic.

The metals tested were characterized by different susceptibility to binding with basic chemicals used for leaching. Significant differences were observed in the percentage of metals in individual fractions leached from the sludge.

The highest percentage of lead, amounting to 26%, was found in hydroxyl fraction III. In other fractions**,** the percentage of lead varied (I**,** 20%**;** II**,** 12%**;** IV**,** 18%**;** V**,** 24%). In terms of binding capacity of lead measured by the content in sewage sludge, individual fractions can be arranged in the following order: III > V > I > IV > II. The tests showed strong binding of lead with iron and manganese oxides and hydroxides. Similar results were also obtained by Scancar et al. (2001) and Rosik-Dulewska et al. (2007), who stated that due to sorption by oxides and hydroxides, lead introduced with sewage sludge into the environment was slightly toxic to plants. Lead content in the residual fraction, unavailable to plants, is also significant, as shown by Renoux et al. (2007) and Garcia-Delgado et al. (2007).

Nickel and cadmium were most strongly bound in the sewage sludge crystal lattice (the remaining part), and their percentage in this fraction was 35% for nickel and 36% for cadmium. On the other hand, they were bound to the smallest extent by the carbonate and hydroxyl fractions. On average, 15% of metals contained in the sludge were bound with organic substances. In terms of nickel binding capacity**,** the individual leached fractions can be arranged in the order: V > I > IV > II > III, and in the case of cadmium**,** in the following order: V > I > II > III > IV. Similar conclusions were reached by Garcia-Delgado et al. (2007), who found that in the residual fraction**,** there was 25–60% nickel. In turn, Wong et al. (2001) showed that nearly 40% of nickel was bound to Mn and Fe oxides. On the other hand, Jamali et al. (2007) found that the largest amounts of cadmium were associated with the fraction bound to Mn and Fe oxides (52%) and with the residual fraction (32%). Only 9.1% of this element was associated with the residual fraction.

The dominant fraction that most strongly bound copper and mercury in sewage sludge was the ion-exchange fraction (I**,** 27% copper**;** I**,** 25% mercury). The metal content was slightly lower in the residual and carbonate fraction: V**,** 24%**;** II**,** 19%**;** and II**,** 22%**;** V**,** 17%**,** respectively. On average, about 16% of copper and mercury were bound to iron and manganese oxides (III) and organic substances (IV). In terms of copper and mercury binding capacity**,** the individual leached fractions can be ordered as follows: I > V > II > IV > III and I > II > III > V > IV. Analysis of the percentage of copper and mercury in individual fractions show**s** that in the sludge**,** they were bound mainly in the first fraction which contained weakly adsorbed and acid-soluble compounds sensitive to pH changes. Garcia-Delgado et al. (2007) showed a different percentage of individual fractions. The authors observed a small amount of copper in the dissolved and ion-exchange form of 0.8–10%**,** and thus showed that copper did not constitute a major phytotoxic hazard when introduced into soil. They found that copper was strongly bound with clay minerals and also precipitated in the form of sulfides, sulfates**,** and carbonates, creating forms with low mobility.

The percentage of zinc, arsenic**,** and chromium in five fractions of sewage sludge was fairly evenly distributed**—**around 22%. Only in the case of organic fraction**,** a slightly smaller percentage of these metals were observed**—**at the level of 15%. Scancar et al. (2001) and Jamali et al. (2007) arrived at slightly different conclusions. They found that chromium in sewage sludge occurred mainly in the residual and organic phase. Chromium was found in small amounts in sewage sludge in the form of carbonates and readily soluble compounds. The low content of chromium in those fractions was most probably due to the presence of organic carbon.

## Conclusions

Determination of total metal content in sewage sludge is used only to assess the sludge contamination. A pool of potentially bio-available elements cannot be determined on this basis. Therefore, it is important to determine the amount of metals bound with the individual components of sewage sludge. Such an assessment can be based on the sequential extraction method (Cai et al. 2007; Fuentes et al. 2008; Lasheen and Ammar 2009; Zhu et al. 2011).

In the study, 15 experiments were conducted to determine the degree of immobilization of selected metals in sewage sludge from the Combined Sewage Treatment Plant in Lodz. The degree of leaching of metals from sewage sludge depended on the type of element and extractant. Copper was the most mobile metal that was at the same time the most bio-available. Mercury and zinc were also mainly associated with mobile fractions which indicate the possibility of their penetration into water. The smallest mobility was characteristic of nickel and cadmium which occurred in significant quantities in bonds with the residual fraction. The form of sludge has a direct influence on the bio-availability of metals contained in sewage sludge. Metals contained in the sludge can be re-released due to changes in pH, dissolved oxygen content, and redox potential. The storage of sludge under various atmospheric conditions, as well as the decomposition of organic substances contained in them with the participation of microorganisms, may cause release of metals from the sludge (Carlson and Morrison 1992).

As a result of the experiments and observations, it can be stated that metals in sewage sludge subjected to various transformations are very difficult to immobilize. The addition of various compounds and substances to the sludge does not affect a radical increase of leaching of the tested elements from the sludge. The sequential analysis performed by the Tessier method leads to a conclusion that despite the addition of calcium oxide in the amount of 1.5 to 75 g per 10 g dm of sewage sludge, the content of all analyzed elements is at the same level. Accordingly, it can be concluded that the addition of calcium oxide and agent supporting the composting process does not affect the retention of metals in sewage sludge.
